# Reply to Green-Gomez et al. Comment on “Richer et al. Night Vision and Carotenoids (NVC): A Randomized Placebo Controlled Clinical Trial on Effects of Carotenoid Supplementation on Night Vision in Older Adults. *Nutrients* 2021, *13*, 3191”

**DOI:** 10.3390/nu14132770

**Published:** 2022-07-05

**Authors:** Stuart Richer, Steven Novil, Taylor Gullett, Avni Dervishi, Sherwin Nassiri, Co Duong, Robert Davis, Pinakin Gunvant Davey

**Affiliations:** 1 Captain James A Lovell Fed Health Care Facility, North Chicago, IL 60064, USA; livonlivon@yahoo.com; 2 Chicago Medical School, Rosalind Franklin University of Medicine and Science, North Chicago, IL 60064, USA; taylor.gullett@my.rfums.org (T.G.); avni.dervishi@my.rfums.org (A.D.); sherwin.nassiri@my.rfums.org (S.N.); co.duong@my.rfums.org (C.D.); 3 Davis Eye Care Associates, Oak Lawn, IL 60453, USA; eyemanage@aol.com; 4 College of Optometry, Western University of Health Sciences, Pomona, CA 91766, USA

We welcome the additional reviews and insights of Green-Gomez, Roche and Nolan [1] concerning our recent publication [2], “Night Vision and Carotenoid (NVC) Study”, and their request for clarification(s) beyond the open triple-peer-review process. Concerning their major issue, this “Intent to Treat” trial, as explained in the text, was initially designed as a randomized double-masked, double-blind, placebo-controlled trial. The planned subsequent analysis of variance and resultant non-homogeneity of variance based upon the disparate differences in age, BMI and % body fat precluded a between-group analysis, as clearly stated in the text and agreed upon during the formal peer-review process. This is the reason why the reviewers and, ultimately, *Nutrients* published our work as a Communication and not as an RCT.

We disagree that there was a “standard expectation” that other variables related to macular pigment MP (i.e., body fat percentage, age and BMI) would not be comparable post randomization. As a principal investigator of three RCTs, this has been my experience having published these peer-reviewed studies within the context of carotenoid supplementation and age-related macular degeneration, albeit with a larger number of subjects in the treatment and placebo groups (see Richer et al.’s age-related macular degeneration (AMD) studies [3,4,5,6]).

## 1. Repeatability of MPOD Measurements

NVC uses heterochromatic flicker photometry. This technology has been adapted for commercial use by a few companies. We used the QuantifEye^®^ device with a clinical protocol that was independently evaluated. Although others have examined the technology, we are only reporting the repeatability of this commercial device in our hands, and not in the hands of an inventor or prototypes. Two studies have observed the repeatability of the device in two separate and unique populations. The mean difference between the measurements of the first and second measurements was 0.01, and the limits of agreement as assessed by Altman and Bland Plot were narrow [7]. The coefficient repeatability was 0.11 to 0.12 and 0.128, respectively [7,8]. These studies only observed primarily naive individuals that were not trained in this technology. In the present study [2], we find that the standard error is the same for both the baseline and final visits for macular pigment (MP) measurements, and it is therefore unlikely that this sample is different or special. We hope this is now clear.

Thank you for pointing out an error in a single data point in Figure 1B [2]. However, the text is correct. The active group graph left eyes show a mean MPOD circa 0.43 d.u. in the left eye at 6 months. The value is actually 0.37 d.u., as stated in the text, and does not change the statistics. The standard error omitted in the text is 0.04.

## 2. Differences in Human Circulatory Anatomy and Aging

Green-Gomez, Roche and Nolan found it highly unusual that the authors claim an improvement in MPOD for one eye (right eye) and not the other (left eye), given the known published intra-ocular symmetry of macular pigment optical density. In addition to the cited reference for this phenomenon in AMD patients, our collective clinical experience suggests that this is not uncommon, as suggested by laterality differences in human circulatory anatomy and aging. Both structural and visual functional differences are observed every day in the clinical setting.

Glare Recovery—The MDD-2 Macular Adaptometer ™ is a commercial clinical device intended to monitor or test individuals for the integrity of central retinal (macular) function and to indicate possible problems by measuring a developing or pre-existing prolongation of photo stress recovery time. The critique, concerning learning effects, does not discount the fact that our NVC-supplemented group showed glare-recovery times twice as rapid after carotenoid supplementation compared with the placebo at six months, in both eyes, compared to baseline (*t*-test *p* = 0.008 R eyes, and *p* = 0.02 L eyes, respectively, post-supplementation at six months). This is not a 10%, 20% or even 50% effect—this is a robust effect and fully consistent with global glare-recovery carotenoid research and Green-Gomez’s, Roche’s and Nolan’s own publications. Their argument is specious—there is nothing unexpected here, in all of the extant research literature involving lutein and the two zeaxanthin isomers.

## 3. Glare, Contrast Improvement, Preferred Luminance

As explained in Section 2.2 of our publication [2] (Instrumentation,)—The Vimetrics Central Vison Analyzer, a device developed by an ophthalmologist, measures the resolution threshold of each eye at fixation within six presentation modules representing varying degrees of contrast reduction under photopic and mesopic luminance conditions that represent real-world vision tasks at presentation times that simulate real-world fixations [9], and, as such, have demonstrated improved correlations with task performance under those conditions compared with chart measurements. Those with visual acuities below 20/100 were not tested or included. Among the group taking the nutrients, resolutions at baseline that mimicked the contrast and luminance conditions of ETDRS charts (22 patients, 44 eyes) ranged from 20/8 to 20/72, while those taking placebo (9 patients, 18 eyes) ranged from 20/8 to 20/44. At six weeks, the supplementation group (42 eyes) demonstrated an average improvement of 0.03 to 0.07 logMAR vision in all modules compared with baseline, while the placebo group (16 eyes) declined from 0.05 to 11 log MAR; the greatest difference occurred in the mesopic modules with the greatest reduction and, in contrast, in the photopic modules with the worst glare—induced contrast reduction. At 6 months, compared with the baseline, among the supplemented group (34 eyes), this declined to only a minimal improvement of only 0.01 log MAR, while the placebo group (16 eyes) demonstrated a worsened acuity from 0.05 log MAR in the high contrast to 0.15 Log MAR among the modules with lower contrasts. At all durations, compared with baseline, the fraction of the eyes with acuities that worsened greater than the reported reproducibility for age level 8 was much greater among the placebo group than among the nutrient-treated group (please see the table to observe the differences approaching statistical significance at the various timelines, as well as those appearing as clinically significant and approaching statistical significance in this series of limited numbers). There is nothing unexpected, based upon the extant carotenoid visual function literature. It is data consistent with Dr. Nolan’s CREST study.

Preferred Luminance—One can determine from a visual inspection of Figure 3 [2] that there is an appreciable difference between placebo and intervention groups in terms of change from baseline to 6 months, right and left eyes compared to the baseline data (*t*-test *p* = 0.02 and 0.03, respectively) using the simple clinical device and the statistical constraints imposed by post-randomization heterogeneity of variance.

## 4. Useful Field of Vision (UFOV) 

Contrary to Green-Gomez’s, Roche’s and Nolan’s assertion of a “practice effect”, the commercial Brain HQ device and algorithm represents the culmination of 30 years of research in neurological science and related medicine, where practice effects are taken into account. Brain HQ was designed by an international team of neuroscientists, led by Michael Merzenich, PhD, a Professor Emeritus in neurophysiology, a member of the National Academy of Sciences, co-inventor of the cochlear implant and Kavli Prize laureate. We trust the results of the autonomous Brain HQ evaluation as no one in our team had the requisite expertise to develop such a tool. Notwithstanding, our research group was the first in the world to present, in 2015, pilot datum suggesting that dietary zeaxanthin(3R, 3″R) had a positive effect in “delayed memory” brain function (*p* < 0.04) in an RCT, where testing was conducted by a licensed-clinic neuropsychologist using the RBANS (Repeatable Battery for Neurological Status) methodology. That datum was presented as a scientific poster organized by Nolan et al., and later published in a peer–reviewed journal [10].

As scientists, we have an obligation to report the data, including the negative results. The NVC study, at great experimental and financial expense, was indeed designed as an RCT and downgraded to a Communication. As pointed out, limited recruitment played an important factor. As the PI, this was the first time that I encountered a situation of “non-homogeneity of variance” post randomization. The *3 Nutrients* reviewers indeed were uncomfortable with the post-randomization outcome and the Editor insisted the work be published as a short Communication. These are the first words above the title in our publication, at the top of the first page.

The positive visual effects of foveal carotenoids include both the principal dietary plant form used in NVC; zeaxanthin (3R, 3″R) and the uncommon and rare marine form meso-zeaxanthin (3R, 3′S) are both available in nature, but differ by orders of magnitude in an actual human diet. Meso-zeaxanthin derived from nutritionally common lutein (primarily) is only found in minute, clinically insignificant quantities from the skin of trout, sardine and salmon. The effect of dietary carotenoids on visual function from the consumption of dietary/supplemental zeaxanthin, or supplemental high-dose meso-zeaxanthin, has been confirmed by the scientists within our group and at Dr. Nolan’s Waterford Institute. Clearly, this is not the issue that Green-Gomez, Roche and Nolan et al. have with our Communication.

We believe that it might be possible that Green-Gomez, Roche and Nolan take issue with the fact that we did not select a formulation containing meso-zeaxanthin. We believe that meso-zeaxanthin is well tolerated and has clinical benefits in robustly raising macular pigment. It also likely has benefits in advanced cases of age-related macular degeneration. However, we urge caution in the universal promotion of meso-zeaxanthin supplementation, in relation to enhancing brain function, without further study [11]. Dr. Nolan’s 2018 study involved fewer patients than our Communication, ignores the fact that the meso-zeaxanthin enantiomer is non-existent in the human brain, and ignores the potential detrimental effect of a supra-physiologic dose on brain function if it were to be prescribed in near-isolation as a major carotenoid for brain function. Three independent groups have criticized their pilot cognitive study, which interestingly lacks a placebo group [12,13,14]. We understand this could be construed as an unfair criticism, as a larger Alzheimer’s study is underway at the Waterford Institute, Ireland. The scientific community awaits their results concerning whether or not meso-zeaxanthin has a future role in brain health and function, beyond that of enhancing central foveal visual function. Regardless, Dr. Nolan has done a remarkable job by increasing the public’s awareness of the vital ocular-retinal importance of all three carotenoids. Our team thanks Green-Gomez, Roche and Nolan for their meticulous analysis of our Communication.

The NVC group has now shown that the major dietary carotenoid “zeaxanthin”, from plants, does have a positive effect on brain function, and this new datum agrees with our previous RCT publication [10]. This is the key new insight provided in our Communication. The NVC team agreed to an open peer-review process. In addition, we believe that we have not overstated the data in our Communication. All NVC peer reviews can be found at *Nutrients* [2]. 
